# ‘As if the soul returns to the body’: affect, stuckedness, and (in)voluntary return to Nicaragua from Spain

**DOI:** 10.1080/1369183X.2024.2377862

**Published:** 2024-08-29

**Authors:** Elise Hjalmarson

**Affiliations:** Department of Anthropology and Sociology, Geneva Graduate Institute, Geneva, Switzerland

**Keywords:** Affect, emotion, choice, migration, return

## Abstract

This article attends to the emotional resonances of ‘stuckedness’ and (in)voluntary return as experienced by Nicaraguan migrants stranded in Spain during the COVID-19 pandemic. Feeling both figuratively and literally trapped in a context of cascading lockdowns, border closures, and travel restrictions, many viewed Spain’s Assisted Voluntary Return and Reintegration (AVRR) programme as offering a choice to ‘freely’ depart Spain – a way to simultaneously leave their distressing circumstances behind while returning to the comfort of ‘home’ and family. Building on recent literature that challenges the basis for participation in such programmes as founded on free, voluntary, and individual decisions, this article contends that, for some, the act of ‘choosing’ to return generates a profound and unexpected emotional response. In the case of a Nicaraguan migrant woman explored in detail here, the execution of said return activates feelings of relief, euphoria, and hope, as well as a renewed sense of self. Nevertheless, her response is temporally and geographically contingent, as returning to Nicaragua via AVRR does not necessarily diminish her desire to remigrate or render her less ‘stuck’. The empirical material that informs this paper was collected through digital ethnography and in-person encounters in both Spain and Nicaragua between 2020 and 2021.

## Introduction

Amanda’s^1^ WhatsApp messages arrived in a steady stream, mimicking the rhythms of a conversation one might have in person: unwitting white text bubbles, light and weightless, incognisant of the emotion they carried across the virtual space and physical distance between us. ‘I brought my two children [here], and now I just want to return to my country … and I feel like a prisoner, unable to go back’, her message read. I shifted anxiously on the couch as I tried to think of how to respond to this person I had never met, an illegalised migrant woman from Nicaragua the same age as me. ‘Sometimes I feel like a failure, at thirty-three years old, living with my mother and my children, and she provides for [us]’, Amanda continued in another message. ‘Suffering is part of life. I hope that it’s temporary. I haven’t seen the light in a few years’.

Amanda and I met by unusual means. In the spring of 2020, during the first wave of the COVID-19 pandemic in Spain, both she and I joined a WhatsApp group of Nicaraguans trying to find their way home. A country that I have visited more times than I can reliably count, I lived in Nicaragua for three years until 2018, when I relocated to Europe to begin my PhD. Then in Spain for my fieldwork, my former Nicaraguan partner and I had purchased flights to his home country in April in a last-ditch effort to escape the escalating sanitary crisis overtaking the continent. Travel, however, eluded us, as it did most of the Nicaraguans in our WhatsApp group and so many millions around the world immobilised by measures to stay the virus’s spread. As the pandemic wore on, our WhatsApp group chatted daily, keeping each other apprised of our travel plans and exchanging news, texts, and multimodal messages of faith and heartache. Stranded in various towns and cities across Spain and confined to our apartments – and despite our vastly different situations and positionalities – our WhatsApp group became a source of emotional resonance. While certainly no substitute for the material support many so desperately needed, together we came to form an ‘emotional community’ (Margrit Pernau in Kumarasinghe 2020) of intimate strangers. Many of us would depart for Nicaragua eventually and, in the months that followed, our WhatsApp group grew steadily smaller as returnees abandoned the chat.

More than a year later, in August 2021, Amanda and I met in person in Nicaragua. She and her children had returned home the year prior via Spain’s Assisted Voluntary Return and Reintegration (AVRR) programme [in Spanish: *Proyectos de Retorno Voluntario Asistido y Reintegración*]. To my initial surprise, Amanda spoke casually about her aspirations to return to Spain. How could she consider migrating again after an experience that resulted in her and her children becoming stranded, so that their only seemingly viable option was to self-deport via assisted ‘voluntary’ return? I wondered. In her physical journey from Nicaragua, to Spain, and back again, what affective journey had she taken that might shed light on her desire to remigrate?

This article attends to the affective dimensions of illegalised migration between Nicaragua and Spain with a focus on aspirations, stuckedness, and return. I favour the term ‘illegalised’ over ‘irregular’ or ‘undocumented’, following Kalir (2019b) and Bauder (2014), for its elucidation of the production of illegality and processes of criminalisation to which migrants are subject by neocolonial border regimes and state authorities and which refute their very right to exist in certain spaces. With ‘stuckedness’, I mobilise Hage’s (2009) notion, which ‘presumes a lack of agency’ (4), whereby the ability to act upon a choice, were such choices available, lies beyond one’s reach. Drawing from a combination of digital ethnography and in-person encounters, I show how, even in the context of the COVID-19 pandemic and the immobilising restrictions that ensued, my interlocutors’ experiences of stuckedness were multifaceted, incorporating profound existential and emotional dimensions. Not only did they express anguish at being stranded in Spain and a longing to travel ‘home’ to Nicaragua, but they also articulated an existential stuckedness – a longing to *realizarse,* to make something of themselves, which was sharpened by their sense of being unable to do so in both Nicaragua and Spain. Such stuckedness, I suggest, as it is experienced in an affective, existential, temporal, and material tangle, contributes to the appeal of AVRR to some illegalised migrants. Although the construction of AVRR in Spain as a free, ‘voluntary’, and individual decision obscures both the structural conditions that engender (im)migrant precarity and the coercion to which many returnees are subject (see Strasser and Sökefeld 2025), and in contrast to the defeat, shame, and humiliation experienced by many returnees in the period leading up to their return (see Barone 2025), in some cases, opting to return can generate feelings of relief, gratification, and euphoria. In short, ‘choosing’ mobility – even in the form of return – and the execution of this decision, can be affectively charged in unexpected ways.

In what follows, I begin by attending to the affects engendered by stuckedness as they circulated digitally in our WhatsApp group. Then, I zoom in to focus on Amanda’s narration of her journey – physical and affective – from Nicaragua, to Spain, and back again. Having returned to Nicaragua, she continued to aspire to a better life abroad: one in which she imagined ‘making something’ more of herself and securing increased opportunities for her children. AVRR may have succeeded in removing Amanda from Spanish territory and generated temporarily relief from the agony of being stranded abroad, but it did little to diminish her desire to migrate once she was again confronted with the socioeconomic situation in Nicaragua and immersed in its growing culture of emigration.

## Affects and emotions in migration research

Across the social sciences, scholarly work on emotions emphasises their circumstantial, contextual, unstable nature, as well as their intimate relationship to questions of power. Emotions are intersubjective, a product of cultural and social worlds, and not just interior, individual psyches. From a sociological standpoint, ‘affect “holds together” our experience of being in the world, in movement, and our relation to space, place, and otherness’ (Glaveanu and Womersley 2021, 629). It is, in this sense, a social glue that quite literally moves us – affecting the decisions we make, the pathways we choose – indeed, the pathways themselves. In a Foucauldian sense, we might say that emotions are powerful, creative, and generative.

Affect and emotion are communicative: they proliferate between and among us, through social relations and networks, via symbols, bodies, and objects, and by way of diverse mediums (Ahmed 2004; Gibbs 2001; Wise and Velayutham 2017). They form a veritable economy, not of goods nor services, but of feelings, moral sentiments, and values (Ahmed 2004; Fassin 2009). Just as affective states are indicated through stern expressions and breaking voices, they can be transmitted via multimodal messaging: in the articulation of one’s feelings, the sending of photos and audio messages, as well as via emojis, GIFs, or forwarded images. In the form of network packets, embodied emotions and affective states are virtually routed through fibreoptic cables, compressing the physical distance between us with the aid of digital media technologies such as, in the case of this research, WhatsApp (see also Stark 2019). With their transnational circulation facilitated by digital technologies and quotidian devices like cell phones – and in stark contrast to people – the expression, performance, and sharing of affects knows no national borders.

The circulation of affects exercises profound influence on the migration experience (Wise and Velayutham 2017) and migration itself is generative of emotion (Carling and Collins 2018). Migration evokes shifts in our affective lives and move us into new and different emotional geographies vis-à-vis changes in our social networks, in the mediums we use to keep in touch with one another, and via acute experiences of belonging and non-belonging – of isolation, exclusion, and segregation. Shifting social relations of power and positionalities as migrants move between social and cultural worlds and regimes of value (see Pine 2014) also result in new affective experiences. Boccagni (n.d.) suggests that the migration experience is characterised by such striking emotions as nostalgia, ambivalence, and hope. In addition to generating profound ‘felt’ responses, migration is evocative of temporal tensions: between past and what was; present, and what is; and the future, or what will or could be (Boccagni, ibid). As the authors in this Special Issue show, experiences of *removal* – used here to convey the continuum between deportation and assisted voluntary return (see Strasser and Sökefeld 2025) – and the ebb and flow of everyday lives under its shadow, are also intensely emotionally charged.

Attention to migrants’ emotions, affective lives, and subjective experiences functions as a ballast to mainstream migration studies, which continues to cast a spotlight on the economic motivations for migration and sideline research on affect and emotions (Boccagni and Baldassar 2015; Halfacree 2004). Mar’s (2005) work suggests that emotions constitute ‘push and pull’ factors, influencing migrant decision-making processes, strategies, and expectations. In his research among the Lebanese diaspora, Hage (2021) describes the desire to migrate as a ‘propelling force’ which operates ‘continuously from within the subject it is affecting’, existentially driving one to migrate in pursuit of a life that is perceived as ‘going somewhere’ (44). While the bulk of migration research continues to focus on outgoing journeys (Aure and Riabova 2020), there are exceptions which incorporate return, whether ‘voluntary’ or otherwise (see Mar 2005; the articles in this issue). Moreover, focussing on the aspirations and desires of racialised Latin American women in the Spanish metropole reveals once more the always gendered nature of migration experiences and the emotions triggered along neocolonial migration routes. This is significant considering the feminisation of not only global migratory movements, but particularly Latin American migration to Spain, as women increasingly spearhead these migration projects (Gil Araújo and González-Fernández 2014).

## Assisted ‘voluntary’ return from Spain

‘Voluntary’ return programmes, as politically distinguished from ‘forced’ deportations, have constituted a key pillar of migration management in Spain since their introduction in 2008 (Kalir 2019a; Webber 2011). Governed by the Ministry of Inclusion, Social Security, and Migration (MISSM) [in Spanish: *Ministerio de Inclusión, Seguridad Social y Migraciones*] and carried out by numerous non-governmental agencies across the country, AVRR accomplished the departure of more than 22,000 migrants between 2009 and 2021 (MISSM 2022). A mode of migrant governance operating along the ‘deportation continuum’ (Kalir and Wissink 2016), voluntary return programmes have been hailed by governments across Europe and marketed to migrants and the agencies that support them as a dignified alternative to deportation (Cleton and Chauvin 2020; Fine and Walters 2021; Kalir 2019a). In the Spanish context, Kalir (2019b) contends that AVRR operates in tandem with forced removals to build out Spain’s ‘deportation apparatus’ (22). And yet, he notes that return counsellors at various non-governmental agencies in Barcelona view their work to support (and encourage) migrants in their return process as humanitarian, revealing an embedded morality which defends AVRR as ‘a benevolent act for those who failed to manage their life in the destination country’ (Kalir 2019a, 57).

Like deportation, however, assisted voluntary return programmes remain a form of retroactive border control operationalised by the state as a form of racialised population management: an extension of its sovereign right to determine whom it includes versus excludes (see Strasser and Sökefeld 2025). Despite the discursive emphasis such programmes presumably place on migrant-led decision-making, then, numerous studies have called into question the degree of ‘voluntariness’ at play in migrants’ cessation to return programmes (Cleton and Chauvin 2020; Fine and Walters 2021, Webber 2011). These works criticise counsellors ‘induction’ of return aspirations (Cleton and Schweitzer 2020), the lack of support extended to asylum seekers (Keith and Schawaf 2018), the economic precarity generated by migrants’ illegalisation (Kalir 2019a), as well as the ‘manipulative’ way that such programmes mobilise essentialised and idealised notions of ‘home’, thus reifying migrants’ acute experiences of exclusion in their destination country (ibid, 56). At the core of such concerns is a consensus that migrants who ‘choose’ to return ‘voluntarily’ often do so when it appears (or is construed to appear) to be their only remaining option.

Laying aside the critique levied by scholars surrounding voluntary return’s inherent deceptiveness, perception among migrants of the programme’s ‘voluntariness’ is arguably vital to its operation. In their troubling of the construction of the ‘voluntary’ nature of return programmes and the soft-power techniques deployed by government and NGO workers in the Netherlands, Cleton and Chauvin (2020) suggest that return counselling which coerces migrant performances of ‘freedom’ and ‘choice’ is integral to the governance of irregular migration. According to the authors:

‘In the case of “voluntary return”, undocumented migrants must be persuaded to leave by themselves *and* convinced that the decision stems from their own choice. This requires not the use of raw force but of incentives that purport to make the desired behaviour objectively sensible and hence interpretable as a product of “agency”’ (Cleton and Chauvin 2020, 300, emphasis in original).

Calculatingly, then, such programmes exploit precarious migrants’ very determination to assert agency and control over their own migration trajectories – to counter their own existential stuckedness – by inviting them to make a choice in a context of formidable constraint. While in the Dutch case, the authors contend that migrants are expected to ‘forcibly perform agency’ (Cleton and Chauvin 2020, 299), in Spain I observed illegalised migrants in considerable distress plead to a return counsellor for acceptance in AVRR as a last resort. In these cases, ‘deservingness’ was heavily weighted, and substantial biographical information was requested from migrants before a decision was made about their eligibility for ‘assistance’. Although distinct, in both cases migrants are expected to work to be accepted into a voluntary return programme, whether by demonstrating that they have exhausted all other options available to them, such as social assistance or informal family supports, or by performing such acute need – a true ‘desire’, if not desperation, to travel – that the return counsellor concedes (for more on migrants’ emotion work, see Strijbosch 2025).

## ‘I wish I were a bird’: stuckedness in Spain

‘Good afternoon, does anyone know how long it takes to get approved for voluntary return?’ I received Amanda’s message in our group chat on a warm morning in June 2020. Another Nicaraguan participant in our chat, Silveria, replied: ‘They aren’t approving any. I applied in January, and they still haven’t given it to me. The other day, I called, and they told me that it’s all paused’. Amanda responded, expanding: ‘I asked at the International Organization for Migration [(IOM)], and they told me that the programmes were running and that, yes, they are receiving applications … where it is paused is at the Red Cross. With support from the Nicaraguan embassy, the IOM is continuing … at least, that’s what they told me … they said that all of my papers were good and that I just had to wait’. While it was sometimes difficult to gauge the reliability of the information being shared, it is noteworthy that the topic of voluntary return emerged regularly and generated much interest, with group members seeking guidance on how to apply or sharing where they had found information. More often, our chat was affectively amorphous: a swirling sea of emotional offloading as members unburdened themselves of their individual despair to a sympathetic, responsive, and ‘hostage’ collective. What united us, after all, was our shared immobilisation by pandemic restrictions and desire to travel to Nicaragua.

While Nicaraguan emigration has increased steadily since the mid-1990s, recent estimates suggest that outward migration is at an all-time high. Between 2015 and 2020, the number of Nicaraguans seeking refuge abroad increased by 2,645 per cent, rising from 2,706 to 74,285 (Migration Data Portal 2021). The United Nations (2020) estimates that more than 100,000 Nicaraguans have fled the country since the 2018 crisis, when peaceful, student-led protests over social security reforms were violently repressed by the national police in conjunction with state military and pro-government mobs, leaving more than 300 people dead and 2000 injured (Amnesty International 2019). The fallout dealt a devastating blow to the country’s steadily expanding economy, an upswing due largely to increasing tourism and direct foreign investment growing under its delicate democratic tradition. With Nicaraguan migrants historically bound for Costa Rica, the United States, or Panama, Spain emerged only recently as a destination of choice. Even so, when compared to the arrivals of Latin Americans from across the region, the number of Nicaraguans migrating through ‘legal’ avenues to Spain remains modest. According to official statistics, some 57,530 Nicaraguans resided in Spain in 2020. By comparison, more than 270,000 Colombians and nearly 190,000 Venezuelans also lived in Spain (INE 2022).

In contrast to travellers from many other Latin American states, Nicaraguan citizens do not require a visa to travel to Spain. Rather, and not unlike passport-holders of privileged Northern countries, Nicaraguans are granted a 90-day visa-free visiting period. Only a sliver of Nicaragua’s modest population is economically positioned to take advantage of this policy, which is increasingly anomalous for the region. Economic impoverishment and the Atlantic Ocean comprise the most obvious deterrents, making migration to elsewhere in Central America, or north to the United States, while exceedingly difficult and dangerous, more plausible nonetheless.

In an affective twist resonant of Lauren Berlant’s (2011) ‘cruel optimism’ when one considers the substantial effort required to journey from Nicaragua to Spain, the emotional turmoil of now being geographically stuck in Spain – during a pandemic, no less – figured prominently in our group chat. The atmospheric mood fluctuated tremendously from day to day as participants responded to evolving circumstances and their own lack of control over their situations (see Perl 2025). Stuckedness, in Hage’s (2009, 2021) sense of the word, was also discursively interwoven through our virtual conversations. ‘I’m desperate and September is so far away, how horrible, and I don’t have work’, wrote Carla, a mother in her fifties, whose children were in Nicaragua. ‘This is anguish, being here and with the uncertainty of not knowing when we will leave’, she continued on another occasion. Reading Carla’s comments, I was struck by her desire for a travel date, which would constitute a hopeful, concrete event to which she could look forward while also marking an end to the suffering she was experiencing as a precarious migrant mother far from home. Uncertainty, and the anxiety it generated, was one of the most emotionally difficult aspects of our predicament. Our shared geographic and existential immobility also prompted group members to articulate reassessments of their migration decisions, valorising all things Nicaraguan – perceived as certain, familiar, and lasting – over those of Spain – rendered less certain, unfamiliar, and fleeting. In what Hage (2021) calls one of the ‘traumatic moments of migration’ (47), the fear of having migrated for nothing and being forever stuck abroad in the unfamiliar was expressed repeatedly. ‘God willing, I’m desperate to be with my children!!’ Carla lamented in our chat. ‘Euros aren’t happiness, happiness is having the family together, even if it means *frijoles* [beans], but we’re happy’. Such reassessments were no doubt influenced by Spain’s dire sanitary situation, the severity of its lockdown, and the exacerbated economic need experienced by migrants as a result.^2^

Like our chat participants, I waited as the days slipped by, my fieldwork on indefinite hold, my flights to Nicaragua repeatedly cancelled and rescheduled for the following month. Despite my very real drive to leave Spain, my own affective journey through lockdown and stuckedness was quite distinct. As a new PhD researcher recently arrived in Spain to work among Latin American migrants, I was navigating emotional landscapes related to my studies that my interlocutors were not. Much of my own anxiety was related to ‘fieldwork failure’: after all, my project was also ‘stuck’, so to speak. Would my leaving Spain be perceived as my having abandoned my field site and research? On the contrary, was this not the ideal moment to showcase my grit and resilience as an incipient field researcher – to find a creative way to get my project ‘moving’? And if I departed Spain now, I wondered, would it not set me back in the relationships of trust I was striving to build with my interlocutors, foregrounding my privilege and breaking the complicity we shared through our mutual (yet distinct) experiences of stuckedness?

For although we were all stranded in Spain and actively looking for a way to Nicaragua – to the warmth that was family, humidity, and *patios traseros*^3^ shaded by lime trees and drying laundry – the situations in which we found ourselves were unmistakably different.^4^ A white, Canadian woman, I was in Madrid for research, and not to work as an *interna* – a live-in caregiver – or to labour in the Spanish sun harvesting watermelons or tree fruit. Not only did my whiteness shield me from the penetrating gaze of police and immigration officials, but my possession of Swiss residency for my doctoral studies ‘legitimised’ my presence in Spain. By contrast, many in our group chat had remained in Spain beyond their visa-free period and would now be subject to detainment and deportation as illegalised migrants (see Kalir 2019b). Several members were vocal about having gone months without work, so the expensive charter flights publicised by some participants were out of the question. With no employment, and therefore no regular income, remittance funds had dried up, conferring the Nicaraguan group members with another moral and emotional burden, a product of their geographic and material stuckedness, which I was spared.

These divergences noted, I nevertheless found myself emotionally embroiled in our chat and affectively invested in migrants’ individual struggles to return. After all, fieldwork is a fundamentally intersubjective experience, and our dialogue, although digital, engendered profound empathy. As a process through which we ‘gain some access, no matter how mitigated that might be, to the embodied subjective experience of another’ (Throop 2010, 772), empathy is fundamental not only to ethnographic fieldwork, but to consensual relationship building between a researcher and her interlocutors. It entails not only the researcher’s desire to understand the lived experiences of others, but others’ desire to narrate their own experiences and be understood (Throop, ibid). While I was attentive to the evolution of our empathic connections in this virtual space, it is noteworthy that I was also cohabiting with my Nicaraguan partner who was himself desperate to return home. Thus, while some of the empirical material I explore here was gathered via digital ethnography, it is mediated by my physical proximity to and emotional enmeshment with my partner, further sensitising me to the predicaments of my interlocutors.^5^

One of the few Nicaraguan men active in our chat, René became an almost daily source of hearsay and encouragement. ‘Hey group, how are you doing today?’, he texted. ‘Don’t get upset, we’re all in this together. We’re all sad and desperate to go back. But trust in God’. Such ‘emotion work’ (Hochschild 1979; Strijbosch 2025), whereby individual members made efforts to affectively steer the group chat, were common. René continued: ‘What I’d like to know is why they keep cancelling [flights] and when we’ll be able to travel. Because many companies are flying from Madrid to Latin America. If you look, you’ll see people arrived in Madrid from Peru’. René’s affective shepherding was met with ‘amens’, ‘God willing’, and expressions of emotional reciprocity, imbuing our chat with a cautious, if volatile, sense of hope. Similar proclamations of emotional solidarity were frequent and affirmed members’ awareness of not only their shared predicament, but also the parallel emotional journeys they were taking.

‘We already knew that the only option was a repatriation flight. And without coming back here, since they treat us like slaves. Me, when I go, I’m not thinking about coming back, God willing’, Silveria texted one afternoon. ‘I wish I were a bird so that I could fly’, she continued. ‘But … we’ve got to accept our situation’. When Amanda shared her acceptance into AVRR in our WhatsApp group later that month, the chat lit up with excitement. ‘Have a good trip’, one member texted. ‘When you arrive in Nicaragua, I ask that you kneel and give honour and glory to Jehovah, for He promises to take care of the foreigner’. Others requested she share the information which led to her acceptance: which organisation would she travel with? How could they also get in touch with them? Poignantly, Amanda’s return was perceived by many in the group, and by Amanda herself at the time, as a result of divine intervention. As more thoroughly discussed by Barone (2025) and Mahar (2025) in this Special Issue, would-be returnees consistently fell back on their faith in god’s will to both make sense of and cope with feelings of helplessness and their own constrained agency.

## ‘Desired’ departures and contentious returns

While I regularly messaged privately with several group members, it was with Amanda that I formed the closest bond. Over months of multimodal chatting, including lengthy audio messages and photo exchanges, Amanda narrated the morose story of her family’s migration from the Nicaraguan capital of Managua to the Spanish town of Murcia in September 2019. A university graduate, Amanda had been working as a primary school teacher, a humbly remunerated albeit respected position that offered coveted predictability. Her husband also had stable work and their two children were attending private schools – a mark of social distinction. She and her husband owned a small house and a car, which they sold to finance their move to Spain. Taken together, the picture Amanda painted of her family’s former life in Nicaragua was one of modest comforts which they had wagered for the possibility of a future life that they perceived as offering increased opportunities for themselves and their children in Spain.

Just ten months after their arrival, Amanda and her children were living with her mother in Murcia. She was broke and desperate, her savings spent, her children depressed. Her husband had used the last of their money to return to Nicaragua alone, abandoning her and their children in Spain – for another woman in Managua, Amanda presumed. Amanda had worked all manner of jobs – caregiving, farming, cleaning – but nothing which had lasted long or paid enough to cover their day-to-day expenses. She had also rejected numerous offers of sex work which she received in response to the ads she was posting on virtual job boards. Amanda told me repeatedly how she longed to return to Nicaragua. ‘It will never be the same to be in your homeland … Here in Spain, I feel that we are worthless’. I asked her to clarify: in what way did she feel worthless? ‘Morally, economically … . Everything’, she replied. ‘Or for the colour of your skin. They always say [when they meet] me, “Ah, you’re really dark. Is everyone like that in your country?”’

In June 2020, just as Spain began to relax its lockdown measures, Amanda and her children were accepted into Spain’s AVRR programme by a local NGO. It covered their bus tickets from her mother’s house in Murcia to Madrid, paid their flights to Nicaragua, and put 450 euros in her pocket. Though not a clear-cut ‘deportation’ in that she was not forcibly detained and removed, neither was her return via AVRR as straightforward as the programme’s name implies. As they prepared to board their much-anticipated flight home, Amanda texted me excitedly. While conscious of disputes over the ‘voluntary’ nature of such programmes, her palpable relief provoked cautious optimism on my part. Almost as if she knew, she would later write me that coming home was ‘an immense joy: as if the soul returns to the body’.

In August 2021, a little over a year after joining the WhatsApp group of returning Nicaraguans, I stepped out of a taxi at the underground entrance to Plaza Inter*,* a crowded shopping mall in the centre of Managua, Nicaragua. Despite her mask, I recognised Amanda instantly from her WhatsApp photos. She sported a white and red mickey mouse t-shirt, her thick dark curls falling past her shoulders. Our in-person reunion was a happy, if slightly awkward, occasion, eased by our yearlong virtual conversations covering all matter of subjects, from food, to feminism, to infidelity. Together we rode the escalator from the parking lot to the shopping mall’s third floor food court and found seats at a small wooden table, where she narrated the hardships she had experienced in Spain, her eventual departure to Nicaragua, and her life since her return. Coming back had been difficult, she told me: it had taken her many months to find a job, during which she and her children had lived above her aunt’s print shop. In the end, she had reunited with her husband and they were now expecting their third child.

Recalling her search for work in Spain as she sipped her coca cola, her pink straw bobbing in the glass soda bottle, Amanda explained: ‘Once they called me for a job, and later they said, no, no, we want a Spanish woman, you need to be Spanish’. Afterwards, she went to work caring for an elderly Spanish woman. ‘When they saw me, they said “*Madre mía*! What a colour! You look like a *morcilla* [Spanish blood sausage].” And at that time, I didn’t know what a *morcilla* was. I still think about this. And I was like, stunned. It sounded to me like they were comparing me to an animal’. Amanda fell into an uncomfortable silence before continuing. Then, ‘I’ve received really offensive comments’, she observed. ‘For example, the first time I went to Spain, I felt *india*^6^, because the *señora* in the house, her daughter, and her granddaughter … they were like, “And over there, in your country? What are the houses like? Are there hospitals? And the schools, what are they like?” And I told them, ‘Look, Nicaragua is different, but we have highways, telephones … ’. Recalling occasions in which she had to defend her dignity or her homeland, she picked up the pace. ‘And one time’, she recalled amusedly, ‘They took me over to the washing machine – it was an old, old washing machine – and they said to me, “Have you seen a washing machine? Do you know what a washing machine is?” And I said, “Of course. In my house, I have a better washing machine then this. It’s digital, with a touch screen.”’ Amanda snickered, clearly proud of how she had handled this situation with her employer and eager to tell me about it. I joined her in a complicit and irrepressible laugh, which felt justified in the face of such petty indignities.

As alluded to by Amanda’s recounting of her experience with her employer, the governance of Latin American labour migration to Spain is inexorably shaped by the politics of development, coloniality, and empire. Research examining the early linkage of race, nation, and belonging in the Iberian context underscores the formation of ‘Spanishness’ as distinct from ‘other’, less desirable groups whose claim on the nation-state could be limited (Feros 2017). Kalir (2019b) mobilises Arendt’s notion of the ‘subject races’ to account for the ways in which the ‘historic logic of managing non-White populations’ (21) is transferred from the colonies to the metropole, perpetuating and extending colonial practices of governmentality to racialised immigrants. Such practices are motivated in no small part by increasing anxieties over the so-called ‘besiegement of Europe’ and ‘reverse colonialism’ as the accelerating displacement of peoples in the so-called Global South contributes to increased immigration to Europe (Hage 2016). Pointing to the growing reliance of global cities on precarious labour from the South, Gil Araújo (2010) contends that colonial configurations of migration flows reconstitute colonial workers as cheap labour in metropolitan centres. This is particularly relevant in the Spanish context, she contends, where colonial technologies of population governance that organised society based on racialised and gendered hierarchies are ‘re-signified by contemporary global capitalism’ (ibid, 180), perpetuating labour market stratification, the homogenisation of diverse Latin American identities, and notions of who is worthy of inclusion in the nation-state.

As the pandemic wore on and her family’s economic situation grew increasingly dire, Amanda’s search for support took on new urgency. ‘When I saw that my mother didn’t have work either and she had her papers, I thought: if she can’t find work, it’s even less likely for me’, she told me. When he found a job in Nicaragua, her husband, from whom she was separated, sent money to her and their children in Spain. ‘He sent *me* money’, she said with a tone of bewilderment. ‘It wasn’t a lot, but enough for milk, bread, all that. And I said to myself, how is it possible that from there, he’s sending me money, when supposedly I’m in a developed country’. She described her ongoing search for support:

‘Everybody said no. Once I didn’t have any milk and so I went back to *caritas*, to the church, to see if they would give me more milk because I didn’t have any, and they said that I had to wait until the next month. And I told them, but I don’t have any *now*! We don’t have any! “The distribution isn’t until next month,” they said, and I was like, fine. I was out of options. And [in the associations] basically, what they always said to me was, “Return then. Just go. Go in peace.” I always said to them, “But help me to work. Help me! I mean, I’ll do whatever, but help me to work.”’

In the Spanish context and keeping the pandemic’s intensification of migrant precariousness in mind, Amanda’s experience is far from unique. Kalir (2019a) contends that portrayals of voluntary return as in migrants’ ‘best interest’ conveniently leaves out that their appeals for institutional assistance in Spain are frequently denied. The refusal of migrants’ requests for support, whether due to policy or a true lack of capacity, ultimately pushes many to ‘choose’ AVRR, further evidence that ‘the motor behind these schemes is people’s desperation, lack of hope, and the harsh situation in which they find themselves’ (Kalir 2019a, 60). Excluded from the formal labour market due to their precarious immigration status and living under the threat of deportation, it is little wonder that migrants experience acute emotional distress which in turn amplifies their longing for the familiarity of ‘home’. Moffette (2018) argues that migrants with irregular status in Spain are maintained ‘in a space of legal liminality’ (156) which he dubs ‘immigration probation’ (172) whereby both the potential for inclusion and the threat of exclusion are always looming close overhead. Consistent with De Genova’s (2002) articulation of everyday deportability, immigration decisions in Spain are thus not pegged to moments or events, such as with pre-emptive visa applications or physical border crossings, but are continuously (re)assessed even after a migrant has entered the state’s sovereign space (Moffette 2018).

‘I visualised myself here [in Nicaragua], and with my children alright, and that gave me hope’, Amanda responded when I asked her what kept her going through the darkest moments in Spain. As contrasting affective valences, hope and despair have emerged together in the last decade as common to the migration experience. Mar’s (2005) conceptualisation of hope as entailing some form of ‘postponement’ is pertinent for those awaiting or anticipating journeys. He argues that to be in an affective ‘hopeful’ state involves accessing ‘a temporal sense of potential, of having a future’ (Mar 2005, 365). In her work on migration from post-socialist Poland, Pine (2014) contends that ‘hope is also always mirrored or shadowed by its opposite, despair’ (S96). Both affects, she suggests, have temporal dimensions, invoking past experiences to imagine a future which helps one overcome present challenges. In Amanda’s words: ‘I thought, at home, I think I have more support, and I can find work’. Then, when she finally arrived:

‘People said, “It’s so great that you came back,” and “there’s nothing like being in your homeland,” or “no matter what, you can always find a solution here.” But others were negative: “What did you come home for? There’s nothing here.” Or they said, “You were fine there, you should have held out.” Or “You have a better future there”. Sure, there’s a better future there, but I don’t have access to it. It’s like – there, I’m a nobody. Nobody’.

In Amanda’s description of the reactions among her network to her arrival in Nicaragua, I heard echoes of her own words to me narrating her return: ‘It’s as if the soul returns to the body’, she had relayed clairvoyantly, when I had texted her to share my own relief and excitement at having finally landed in Managua. After all, by her own accounts, her brief spell in Spain as an illegalised migrant had stripped her of her sense of self – generating a worthlessness not spontaneously evoked, but rather brought on by her ‘produced illegalisation’ (Tecca 2025) and keen awareness of her exclusion from social and political arenas reserved for Spanish residents and nationals. Amanda had felt herself a failure, not only as a migrant, but as a mother, daughter, and educated woman with ambitions for her future. Their situation became so desperate that Amanda’s estranged husband had sent her money from Nicaragua, inversing the conventional flow of remittances from North to South and evoking feelings of shame, embarrassment, and powerlessness. Despite the considerable suffering she experienced, Amanda offered:

‘I prefer to be here [in Nicaragua], but if another opportunity were to arise, and they were to tell me that the situation had changed, that there were possibilities to migrate and I’d have a future, I would do it. I would do it, because I want a future and I see the conditions of this country. I’m ok, but I know that it isn’t enough, and I want my kids to have access to something else … ’

While Amanda did not specify how precisely she hoped that ‘the situation’ would change, her concern with finding dignified employment that would allow her to support herself and her children would require a work permit and Spanish residency – ‘possibilities to migrate’, to borrow her words, that would render her neither illegalised nor precarious. Thus, as in Tecca’s (2025) article, Amanda wished to remigrate under conditions that would grant her access to the future she imagined for herself, but which had until now remained beyond her grasp.

While at times difficult to comprehend, Amanda’s cautious hope served the affective purpose of providing her with a future horizon brighter than the one she faced in Nicaragua. Also following Hage (2009), Pettit and Ruijtenberg (2020) suggest that the possibility of migration serves as an existential counterweight for stuckedness by nurturing the hope that one’s life is going somewhere, despite migration’s frequent failure to live up to its promises. Speaking with Amanda, I was struck by the parallels between our virtual chats when she was stranded in Spain and our in-person conversations now that she had returned and felt stuck in Nicaragua. Returning via AVRR may have alleviated the immediate emotional suffering she was experiencing abroad – even generated feelings of hope, joy, and relief – but the situation to which she had returned was unchanged, if not worse. Moreover, Nicaragua’s intensifying culture of emigration whereby individual progress and success are associated with building a life abroad meant that Amanda received a mixed reception from her friends and family upon her return, with many unable to comprehend why she would have chosen to return. Perhaps also influenced in part by their responses, she continued to pin her hopes for a better future on migration abroad.

## Conclusion

Within critical migration and border studies, one answer to the question of how many times someone will attempt to cross a border that stands between them and their notion of a ‘better future’ is ‘until they make it across’. But having reached their destination, illegalised migrants face tremendous obstacles to achieving that future. The greatest of these are structural: the production of precariousness and illegality; migrants’ social banishment, economic exclusion, and the racism underlying the dehumanising treatment, workplace exploitation, and housing insecurity experienced by so many. Migrants’ experiences of these injustices are profoundly emotional generative: of anguish, fear, worthlessness, and despair. Upon encountering such barriers, an existential stuckedness sets in as migrants struggle to cultivate the life they had envisioned.

In the pandemic context of lockdowns, border closures, and travel bans, to be ‘stuck’ has clear geographic connotations. Yet following Hage (2009), I have contended that illegalised migrants, stonewalled from achieving their aspirations in Spain, also experience a form of existential and affective ‘stuckedness’ that extends beyond the pandemic context. In such circumstances, Spain’s AVRR programme offers what seems to constitute a choice where few exist: an escape hatch from a migration project judged futile; an invitation to return to the known and familiar, couched in agentic language which frames transnational mobility in the form of ‘soft’ deportation or removal as a ‘choice’. I have suggested that, notwithstanding the power asymmetries and manipulation involved in such programmes, the act of choosing to participate in AVRR and the execution of one’s return can produce a powerful affective response. Indeed, ‘choice’ itself, irrespective of what is being ‘grasped’ (Hage 2009, 4), may illicit unpredictable affects. Just as AVRR preys on migrants’ fears that, despite their migration, their lives are not truly ‘going anywhere’, choosing to return (or be removed) can provoke ‘good’ feelings. In Amanda’s narration of her initial arrival in Nicaragua, these feelings included palpable relief, excitement, gratitude, and renewed hope for the future. Such feelings were, for the most part, temporary: after her return, Amanda was once again immersed in a culture that valorises emigration as a strategy to escape Nicaragua’s dire socioeconomic situation. The renewed sense of stuckedness she experienced with the rekindling of her migration aspirations, tied as they were to the potential for achieving a subjectively better future elsewhere, then appears less enigmatic. Notably, attention to Amanda’s emotional life not only transformed my understanding of her migration experience, but it was through my enduring ethnographic engagement with her across multiple sites, initially by virtual means and eventually in person, that the diverse contingent affects both generated by and generating her removal were rendered visible (see Strasser and Sökefeld 2025).

Whether Amanda will embark on yet another transnational journey remains to be seen. In the meantime, the desire she articulated to migrate again, albeit under conditions that would grant her access to the possibilities she longed for, echoes popular demands from within Spain to deepen migrant inclusion. In recognition of not only the essential labour many migrants perform and the tremendous hardships they faced during the pandemic, but also of the cruel consequences of illegalisation for their daily lives, pro-migrant, anti-racist movements such as *Regularización Ya* [Regularisation Now] have called for the extraordinary and permanent regularisation of the approximately 500,000 migrants with irregular status across the country. If approved, its Popular Legislative Initiative [in Spanish: *Initiativa Legislativa Popular*] backed by more than 800 organisations and recently presented to the Spanish Deputy Congress (Sánchez 2023) would mark a watershed moment in the lives of illegalised migrants in Spain and lay before them a multitude of choices – currently reserved for residents alone – far beyond what is offered by AVRR.

## Notes

1 To preserve confidentiality, all participants have been assigned pseudonyms.

2 In stark contrast to Spain’s response, the Ortega regime in Nicaragua greeted the pandemic’s first waves with widespread denial and by restricting the sanitary initiatives of non-governmental agencies in the country (Pearson, Prado, and Colburn 2020). While not a topic of conversation which surfaced explicitly in our WhatsApp group, the juxtaposition of Spain’s confinement with Nicaragua as a place of ‘freedom’ formed an affective bedrock which underlay much related chat content.

3 Literally, ‘back patios’. Typically, these are uncovered outdoor spaces with high concrete walls and limited manicuring, often containing fruit trees, concrete washbasins, and clotheslines.

4 Here I wish to emphasise both the more conspicuous distinctions that can be drawn between myself as researcher and my interlocutors, as well as the distinctions that must necessarily be drawn between the interlocutors themselves who formed a heterogeneous group. My treatment of the WhatsApp participants as a group and references to my ‘Nicaraguan interlocutors’ is thus not intended to obscure their divergent experiences or distinct positionalities.

5 Importantly, it is not my aim to conflate my partner’s feelings, or my own impressions of my interlocutors’ emotional expressiveness, with my interlocutors’ internal feelings. Rather, I convey as best I can, in a manner undoubtedly mediated by my own emotional experiences, what my interlocutors revealed to me of their embodied emotional experiences.

6 Translating as ‘Indian’, the term refers derogatively to an Indigenous Nicaraguan, and is used to imply that one is backwards, uneducated, and uncivilised.
